# Emergency Physician and Emergency Nurse Communication in the Emergency Department: A Mixed-methods Study

**DOI:** 10.5811/westjem.48511

**Published:** 2026-01-10

**Authors:** David C. Jones, Jeffrey Phillips, Amanda Graveson, Lindsey Hrizuk, Nichole Meuwissen, Evan Alldredge, Matthew Loxton, Esther Choo

**Affiliations:** * Oregon Health & Science University, Department of Emergency Medicine, Portland, Oregon; † Cleveland Clinic, Department of Surgery, Cleveland, Ohio; ‡ University of Kansas Health System, Department of Emergency Medicine, Kansas City, Kansas; § University Health, Department of Emergency Medicine, San Antonio, Texas; || Legacy Emmanuel Hospital, Anesthesia Critical Care, Portland, Oregon; # Blue Faery: The Adrienne Wilson Liver Cancer Association, Birmingham, Alabama

## Abstract

**Introduction:**

The emergency department (ED) is a setting where communication occurs often and with potential consequences for patient care. In this study we sought to determine nurse and physician perspectives on the nature and implications of effective and ineffective communication in the ED.

**Methods:**

We used a mixed-methods design, including an online survey followed by in-person focus groups with emergency nurses (EN) and emergency physicians (EP). Participants were recruited through email listserves to emergency staff at four hospitals. We integrated quantitative survey results with focus-group themes.

**Results:**

A total of 115 eligible ENs and EPs completed the initial questionnaire (50% response rate from ENs, 65% response rate from EPs). Responses from nurses and physicians were similar; both noted that poor communication is frequent, adversely affects patient care and ED function, affects trust, particularly between individuals, and that non-verbal communication behaviors affect team communication. In the focus groups (consisting of 18 EPs and 17 ENs), six themes emerged: 1) Situations, built physical environment, and medium of communications all impact quality of communication; 2) core elements of desired professional communication include respect and attention, often conveyed through non-verbal behaviors; 3) poor communication begets poor communication and influences interpersonal relationships; 4) effective communication is seen as fundamental to patient care but also has impacts beyond patient care; 5) clinician gender and gender dyads influence communication dynamics; and 6) participants were able to identify learning activities and techniques for effective communication.

**Conclusion:**

Emergency nurses and physicians across four EDs described failures of communication as both frequent and significant to patient care. This study identified characteristics of effective communication, complex factors influencing communication, and emphasized the whole-team impact of communication quality.

## INTRODUCTION

Interpersonal communication is a fundamental skill in emergency care.^1^ Optimal care in high-acuity, high-volume settings requires consistent, active communication within the team. Communication is subject to bias and error; thus, optimizing it plays a cross-cutting role in patient safety and quality of care.^2^,^3^ Lapses in communication can negatively affect the medical team’s functionality, strain individual clinicians resulting in burnout, and are detrimental to patient care.^4^,^5^ While teamwork and collaboration are emphasized in the emergency department (ED),^6^,^7^ particularly in resuscitation, routine communications between emergency physicians (EP) and emergency nurses (EN) in non-critical situations is teamwork that has not been studied in depth yet potentially affects all interactions.

The literature has examined challenges with nurse-physician communication, finding that multidisciplinary teams’ communication can affect patient care positively or negatively.^8^ In a 2012 review of ED communication, when communication was centered on patient care, individuals were prepared, prompt, and minimized interruptions. The review identified challenges to effective communication, including nurses and physicians misunderstanding each other’s roles, hierarchical environments, communication style (descriptive vs succinct), interruptions, and stressful working environments.^8^ Additional literature identified factors associated with effective nurses-physician communication, including mutual understanding, trust, respect, and collaborative attitudes, and factors inhibiting effective communication, including lack of communication, indirect modes of communication (eg, via the electronic health record [EHR] system), insufficient information, selective communication, language, and cultural factors.^9^,^10^

While there is literature addressing general physician/nurse communication,^11^ little is known about direct interactions specific to the ED. Studies related to ED communication focused on areas tangential to effective communication among EN/EP team members, for example, physician-patient interactions,^12^ physician-consultant exchanges,^13^,^14^ or handoffs.^15^,^16^ A better understanding of EN/EP communication—including ineffective communication—could facilitate intervention development to systematically identify and ameliorate such interactions, potentially decreasing errors, harm, costs, and stress on healthcare staff. Our objective in this study was to explore effective and ineffective communication, the influence of individual and system factors on communication, and the perceived impact of communication practices on quality and efficiency of care.

## METHODS

### Study Design

This was a mixed-methods study of EPs and ENs with an explanatory sequential design, using focus-group data to provide context and deeper insights into survey responses.^17^ Participants completed an online survey to identify general types and frequency of communication challenges and facilitators and then attended focus groups further exploring and characterizing the quantitative findings.

### Study Participants and Setting

We defined ENs as registered nurses primarily employed in ED. EPs were defined as physicians who were in their third year of training or had completed training in emergency medicine (EM). We included ENs/EPs practicing at least half of their employment time in one of four EDs: an urban, academic Level I trauma center (40,000 visits/year) that is the primary teaching site for a three-year residency program; an urban community ED 10 miles away (35,000 visits/year); a suburban community ED approximately 20 miles away (9,000 visits/year); and a rural ED 98 miles away (14,000 visits/year). Exclusion criteria were less than two years of experience in emergency medicine and working exclusively in a pediatric ED. These exclusion criteria were chosen to ensure sufficient experience in EM. Clinicians working solely in the pediatric ED were excluded because the environment —a small, stable group of physicians and nurses, low-volume practice, and small physical footprint—is sufficiently distinct that we felt issues raised might not be generalizable to other EDs.

**Population Health Research Capsule**

What do we already know about this issue?

*Team communication in emergency medicine is critical to quality care. Breakdowns in communication lead to errors, breakdowns in team dynamics, and patient harm*.

What was the research question?

*How often do communication breakdowns occur and how can we counteract them?*

What was the major finding of the study?

*90% of emergency clinicians feel poor communication occurs during every shift, and 95% believe it adversely affects patient care*.

How does this improve population health?

*This study identified characteristics of effective communication, complex factors influencing communication, and emphasized the whole-department impact of communication quality*.

### Data Collection Tool

We developed a novel questionnaire to examine the types, frequency, and impact of potential communication gaps between healthcare clinicians caring for ED patients. The initial email questionnaire was based on a literature review of existing health communication literature and observed experiences of study team members. Review details are in Appendix A. Questions were guided by the Input-Mediator-Output framework, conceptualizing how information flows through systems.^18^ It has been applied to healthcare teamwork and aligns with hospital communication processes, considering clinical information and decision-making (inputs), staff and communication systems (mediator), and team impacts and patient outcomes (output). The questionnaire also included basic information about participants, including role/degree, practice experience, and demographic data.

The collaborative team reviewed and revised the questionnaire through an iterative process of face validity testing with structured feedback responses by individuals who met inclusion criteria but were excluded from the final survey population. The trial group responded to the questionnaire twice, one week apart, to assess reliability, and responses were identical 94% of the time. The final questionnaire (Appendix B) had six items assessing frequency of poor or ineffective communication, impact of communication on trust among colleagues, ED clinical team functioning, patient care, and the effect of non-verbal behaviors. Questions were single-answer multiple choice questions.

An initial focus-group discussion guide was created using the same framework and literature review, designed to align with and provide depth and context to survey data. The guide was reviewed by team members and evaluated for face validity as described above for survey development. The final interview guide (Appendix C) had six topics, with suggested prompts and probes, exploring the importance of EN/EP communication, needed and desired information to be exchanged, good communication techniques, poor communication techniques, identification of communication gaps, and behaviors that contribute to communication.

### Data Collection

ENs/EPs in four different EDs were invited to participate in the questionnaire via internal email listservs distributed by ED nurse managers and medical directors. Invitees received up to four requests to complete the survey through their work emails. Respondents meeting inclusion criteria were invited to take part in the full questionnaire. Those who completed the questionnaire were invited to participate in focus groups with EPs and ENs to characterize communication gaps further. Questionnaire responses were stored anonymously to mask responses from focus-group leaders. Questionnaire responses led to discipline-based focus groups. Two trained interviewers moderated focus groups, one a non-clinical co moderator (in all groups), for consistency, and one a co-moderator from the aligned discipline (nursing or medicine [DJ, NM, LH]), to ensure clinical context, language, and nuance were conveyed and captured. Moderators used prompts and probes to elicit and clarify information. Focus groups lasted 60–90 minutes. Patient safety was defined as accurate clinical assessments, correct treatment interventions, and disposition plans that minimize harm, but specific incidents were left to participant opinion.

Focus groups were recorded on digital audio recording software, transcribed verbatim using a human transcription service (TranscriptionPuppy, Miami, FL), and entered into qualitative data management software (MaxQDA 2018). Transcripts were anonymized by replacing names with numerical identifiers to maintain theme continuity from individuals while protecting their identities.

### Data Analysis

For demographic information and survey results, we calculated summary statistics, stratified by role (EP/EN).

We used thematic analysis for focus group results entailing identifying recurring patterns, themes, and categories within the data relating to the nature and impact of different communication behaviors in the ED. Preliminary codes were created from major topic headings in the interview guide and refined by iterative, repeated transcript analysis by the research team, resulting in creation of an initial codebook with definitions. Research team members reviewed and edited the document together to ensure there was a shared understanding of codes and to identify any needed revisions. Research team members included both EPs and nurses, as well as a medical student and a non-physician team member without experience in ED research or practice. During study planning, data collection, and analysis, we included individual reflection and team discussion of the positionality of the ED team members with respect to the data and power dynamics within the team. An integrated codebook, consisting of mutually agreed-upon codes, was entered into the MaxQDA database with the final version of each transcript. Two independent coders (JP and AG) applied the coding scheme to each transcript. Notes taken during interviews were reviewed to understand conversation context, tone, and interactions among participants. Coding discrepancies or ambiguities were resolved through discussion during full team meetings.

We summarized major themes and subthemes into an initial thematic framework based on commonalities, shared meanings, and applicability to the research aims, including alignment with broad survey topics. The study team collaboratively finalized themes and selected illustrative quotes representing the range of responses relevant to each theme. Data interpretation differences were addressed through discussion and re-review of transcripts and coded materials, ensuring that quotes were understood fully and in context.

### Ethical Approval and Competing Interests

The Oregon Health & Science University (OHSU) Review Board exempted this study. The authors have no competing interests. This study received institutional funding from the OHSU Department of Emergency Medicine.

## RESULTS

There were 146 survey respondents, with 115 eligible participants, including 81 ENs (of 164 possible EN participants, 50% response rate) and 34 EPs (of 52 possible EPs, 65% response rate), who completed the entire survey. Table 1 lists EP/EN demographics. Each discipline was asked the same six questions, with the results in Table 2. Although the surveys were sent to four hospitals, several EPs/ENs worked at multiple sites. Therefore, participants represented nine hospitals across seven healthcare systems.

A total of 18 EPs and 17 ENs agreed to participate in focus groups separated by professional discipline. Seven focus groups (three EP and four EN groups) were conducted. Some focus group participants and facilitators/authors knew each other from working together.

### Survey Results

Table 2 summarizes survey results. Overall, ENs/EPs felt poor or ineffective communication occurs commonly, most agreeing that such communication occurs 1–4 times per shift. Further, both disciplines agreed that these communication challenges adversely affected patient care at least sometimes and prevented the medical team from functioning well. Poor EN/EP communication was felt to affect both the trust afforded the individual dyad and “sometimes” the trust afforded the entire discipline. Non-verbal behaviors were felt to impact communication interactions.

### Focus Group Results

Qualitative analysis identified six major themes, summarized along with representative quotes in Table 3.

#### Theme 1: Situations, built physical environment, and medium of communications all impact quality of communication

Participants observed that certain clinical situations tend to be associated with more effective communication, for example, acute resuscitations where all parties are physically and temporally close, while other situations tend to have poorer communication, notably patients boarding for prolonged periods in the ED with transitions between multiple care teams. Further, situational elements influence consistent communication. For example, if a group of ENs is talking, the EP may be less likely to interrupt and communicate with the specific EN about a specific patient. Both disciplines identified preferences with regard to means of communication. Face-to-face communication was most appreciated. Common work areas facilitated communication through proximity and presence. Telephone communication was felt to be the second-best option, although concerns about inopportune timing of calls and task interruption from calls were raised by both disciplines. Electronic communications (chart communications, nursing orders, or “sticky notes”) through the EHR had ease-of-use advantages and potential for positive communication; however, it could also cause conflict. Electronic communications were felt to have the greatest risk of misinterpretation, as well as the highest risk of being ignored or missed by the receiving party. However, it was felt to be a non-intrusive way to communicate simple messages (for example, “labs are back,” or “pain medicine ordered”). Other technologies (video chat, voicemail) were not thought to be practical in the ED.

#### Theme 2: Core elements of desired professional communication include respect, closed-loop communication, and attention, often conveyed through non-verbal behaviors

Participants identified communication components important for effective information transfer and the EN/EP relationship, including specific verbal and non-verbal actions. Conveying respect for colleagues, recognizing the training and experience of the other person, explicitly acknowledging receipt of communication, and physically demonstrating listening were mentioned by both disciplines as necessary components communication. Nonverbal behaviors were noted to impact the quality of communication. Making eye contact, turning toward a speaker, and nodding at appropriate moments to acknowledge active listening were positive behaviors that conveyed respect and strengthened the relationship. Conversely, behaviors such as arm crossing, continuing typing while someone is talking, and not making eye contact or eye-rolling were noted to have negative impacts on the EP/EN relationship. Traditional medical hierarchy has physicians at the top and nurses in subordinate roles.^14^ Participants agreed that although this traditional dynamic is changing, persisting manifestations such as lack of respect toward nurses from physicians can make effective communication difficult for nurses.^14^ Participants agreed both parties share communication responsibility and that both disciplines have challenges initiating and sustaining good communication with one another.

#### Theme 3: Poor communication begets poor communication in later interactions

Participants reflected that the absence of Theme 2 critical elements may beget communication hesitance, resulting in poor communication and team disconnect. Participants shared that ENs with information may hesitate to relay the information to an EP if there has been a prior negative interaction with that EP, or with physicians in general. Additionally, EPs may not share information they have with ENs if they do not believe that there is an immediate need for the EN to know that detail, losing the opportunity to exchange further information.

#### Theme 4: Effective communication is seen as fundamental to patient care but also has impacts beyond patient care

Both disciplines agreed communication is critically important for team dynamics that translate into the quality of care that patients receive. Participants described key roles of effective communication, including creating a shared understanding of the patient’s clinical status and care plan, guiding their clinical actions, and affecting their ability to communicate with patients and families. Participants expressed that poor communication leads to decrements in care, not just for the immediate patient, but potentially for all patients cared for by the EN/EP dyad. Participants also noted that perceived positive or negative communication had spillover effects, impacting care beyond any single case, as it affects the “feeling” of the entire department.

#### Theme 5: Clinician gender and gender dyads influence communication dynamics

Age was downplayed as a positive or negative impact factor with experienced EPs/ENs having good and bad communication habits in equal portions. Experience working together did tend to lead toward positive communication. Sex seemed to play a role in communication dynamics. The greatest communication conflict (noted by both disciplines) involved communication in female/female physician/nurse dyads. Male/male communication dyads had the least perceived conflict, with males often finding commonalities to discuss or bond over that are outside patient care and medical work. Male/female EN/EP or EP/EN combinations were not perceived to have excessive communication challenges.

#### Theme 6: Techniques for effective communication can be implemented

Participants identified structured learning activities and techniques that might contribute to more effective communication behaviors in the ED. Activities mentioned include programs like TeamSTEPPS or American Heart Association life support classes. Specific techniques mentioned include closed-loop communication, creating a shared mental model, and asking clarifying questions. However, as identified under Theme 1, such learning activities are focused on high-acuity situations (eg, resuscitations) and not routine care of more stable and long-term patients.

## DISCUSSION

Our multisite, exploratory mixed-methods study of EPs/Ens communication demonstrated that both disciplines consider communication vital to high-quality care, yet it is often inadequate. ENs/EPs share views on the occurrence of communication failures and impact on patient care, shared perspectives on characteristics of effective communication and responsibility for communicating well, effect of communication on the team’s “feel” at work, and the need for better communication outside episodic resuscitation care (where existing communication training tends to focus). Most participants felt communication gaps had a significant impact on patients and team dynamics and that non-verbal communication affects the quality and perception of communication. The persistence of hierarchies and lack of respect and recognition of knowledge and experience were felt more heavily among ENs.

Current literature recognizes communication as vital to patient care, and poor or lacking communication can have detrimental effects on patient outcomes.^19^,^20^ Our study corroborated previous findings in other settings. We identified times when communication was highly effective, with positive returns in care, including in-person episodes of acute team-based care. In-person communication continued to be the most common and preferred means of communicating, despite advances in technologies.^21^

Respondents observed that sex and nurse/physician sex dyads do affect inter-disciplinary communication, consistent with prior literature. In a study of nurse and resident physicians, Manchada et al found that female/female EN/EP interactions can be strained,^2^ more than male/male (EN/EP) or female/male EN/EP (or EP/EN) relationships. They found that ENs and resident EPs often had differences in communication perception, with nurses feeling more comfortable asking for clarification from female resident EPs, while female resident EPs perceived questioning their care in a way male emergency resident physicians did not experience. While we did not purposefully over-sample or have built-in probes about sex dynamics, we note that our sample was disproportionately women, potentially creating an environment where such issues might be more comfortably verbalized.

In the questionnaire, both disciplines indicated poor communication affects the trust of the individual and sometimes their discipline as a whole; within focus groups, participants elaborated how poor communication initiates a cascade of effects on patient safety, team function, and interpersonal relationships. On a positive note, respondents identified existing trainings and learnable communication behaviors, suggesting teams can improve identified communication issues. Further study is needed to evaluate how to advance behaviors enhancing EN/EP communication and relationships, how the physical space and technology can be optimized to facilitate effective communication, and optimal training to establish and sustain effective communication.

## LIMITATIONS

There are limitations to our study. Due to the anonymized nature of the survey, we were unable to confirm there were no repeat responders. Among those who self-identified for the focus groups, there were no repeat responders. Response rates were modest, potentially limiting generalizability. Surveying within the study team’s own group may have biased the sample in either direction (eg, encouraging or discouraging participation due to familiarity); anonymity guarded against concerns of privacy and confidentiality. However, the sample was robust compared to other studies of its kind, and the heterogeneity of responses suggests capture of a wide range of experience and perspectives. While our sample had heterogeneity in terms of setting, the data represents a single state, and findings could be limited to this region. Given the universality of many aspects of emergency care in the US, we suspect few observations were uniquely local. Our sample was not racial or ethnically diverse; while it is representative of the workforce demographics in this study, it limits the generalizability and identification of intersectional elements of communication in the ED setting. The communication impact of sex dynamics was an unexpected finding; however, only traditional male and female roles were explored, and non-binary gender identities were not discussed.

## CONCLUSION

Emergency nurses and emergency physicians across four EDs described communication failures as common and significant to patient care. This study identified characteristics and modalities of effective communication, complex factors influencing communication, including clinician sex dynamics and type of care, and emphasized the impact on the whole department impact of communication quality.

## Supplementary Information



## Figures and Tables

**Table 1:** Demographics of clinicians responding to a survey regarding communication practices between emergency physicians and emergency nurses.

Category	Emergency physicians N=34	Emergency nurses N=81
Sex		
Male	14 (41.2%)	14 (17.3%)
Female	20 (58.5%)	65 (80.3%)
Prefer not to answer	0 (0%)	2 (2.5%)
Age		
<35	13 (38.2%)	31 (38.3%)
35–<45	15 (44.1%)	25 (30.9%)
45–<55	2 (5.9%)	13 (16.1%)
55+	4 (11.8%)	12 (14.8%)
Experience		
2–<5 years	13 (38.2%)	30 (37.1%)
5–<10 years	12 (35.3%)	16 (19.8%)
10–<15	3 (8.8%)	10 (12.4%)
15–<20	1 (2.9%)	12 (14.8%)
>20 years	5 (14.7%)	13 (16.1%)
Race		
White	27 (79.4%)	73 (90.1%)
Asian	4 (11.8%)	0 (0%)
Prefer not to identify	3 (8.8%)	8 (9.9%)
Ethnicity		
Hispanic or Latino	1 (2.9%)	3 (3.7%)
Non-Hispanic or Latino	27 (79.4%)	72 (88.9%)
Prefer not to identify	6 (17.7%)	6 (7.4%)
Primary practice type		
Urban academic	23 (67.7%)	42 (51.9%)
Urban/suburban	8 (23.6%)	26 (32.1%)
Rural community	3 (8.7%)	13 (16.0%)

**Table 2:** Questionnaire responses by clinicians responding to a survey regarding communication practices between emergency physicians and nurses.

	Emergency physicians (N=34)	Emergency nurses (N=81)
How often on shift do you experience poor or ineffective emergency physician-nurse (EP/EN)?		
Never	1 (3.0%)	5 (6.49%)
1–4 times per shift	26 (78.8%)	60 (77.9%)
5–10 times per shift	6 (18.2%)	11 (14.9%)
>10 times per shift	0 (0.0%)	1 (1.3%)
How often does poor or ineffective EP/EN communication adversely affect patient care?		
Never	0 (0.0%)	6 (7.8%)
Sometimes	27 (81.8%)	53 (68.8%)
Often	5 (15.2%)	17 (22.1%)
Always	1 (3.0%)	1 (1.3%)
How often does poor or ineffective EP/EN communication prevent the ED clinical team from functioning?		
Never	0 (0.0%)	4 (5.2%)
Sometimes	14 (42.2%)	39 (50.7%)
Often	16 (48.5%)	31 (40.3%)
Always	3 (9.0%)	3 (3.9%)
How often does poor or ineffective EP/EN communication affect the trust you place in the individual?		
Never	2 (6.0%)	9 (11.7%)
Sometimes	19 (57.6%)	37 (48.1%)
Often	11 (33.3%)	29 (37.7%)
Always	1 (3.0%)	2 (2.6%)
How often does poor or ineffective EP/EN communication affect the trust you place in most EPs/ENs?		
Never	15 (45.5%)	32 (41.60%)
Sometimes	17 (51.5%)	37 (48.1%)
Often	1 (3.0%)	7 (9.1%)
Always	0 (0.0%)	1 (1.3%)
How much are non-verbal behaviors a factor in effective team communication in the ED?		
Never	0 (0.0%)	0 (0.0%)
Sometimes	7 (21.2%)	43 (55.8%)
Often	23 (69.7%)	27 (35.1%)
Always	3 (9.0%)	7 (9.1%)

*EN*, emergency nurse; *EP*, emergency physician; *ED*, emergency department.

**Table 3:** Summary of themes with illustrative quotes from focus groups examining communication between emergency physicians and emergency nurses.

Theme	Illustrative quote(s)
1: Situations, built physical environment, and medium of communications all impact quality of communication.	Physician 5 – “It is a challenge where the departments are set up [with] nurses and the doctors [seated] in separate areas … doctors’ room on the side and then like the nurses’ on the back hallway … it’s hard to communicate with the nurses [because] I can’t just look up and see them.”
2: Core elements of desired professional communication include respect, closed-loop communication, and attention, often conveyed through non-verbal behaviors.	Physician 3 – “making eye contact and facing the person that you’re talking with is a great concept” Nurse 4 – “stopping and turning away from the computer for a second if you need to be heard is a clear sign of respect.” Physician 4 – “If someone is talking to you, give them the respect of looking at them, [and] turning around.”
3: Poor communication begets poor communication in later interactions	Nurse 2 - “It was told to me that I was completely off base... I should go away. Maybe it would [be] safer and better for the patient if me and this doctor stopped talking to each other.” Nurse 2 - “There’s people whose ego is there and if they have a nurse telling them what to do, then they absolutely will not do what is being said to the point of where safety is completely compromised. I need to stop talking to this person, they need to figure it out on their own, because talking to them now is actually making them dig in more.”
4: Effective communication is seen as fundamental to patient care but also has impacts beyond patient care	Nurse 1 - “Communication... is… the most important [thing] in regard to patient care. More so than actual knowledge... simply because if you work with someone who isn’t a great communicator then it’s really hard to fully understand what’s expected next of you and [to] communicate what we’re seeing to a physician or a team that isn’t otherwise at bedside.” Nurse 5 - “Her [physician] coming up to me and explaining why she wants to go this way -- or that way -- feels inviting for me to contribute, and to have open communication with the patient.” Physician 1 - “Team communication is critical to our job and it’s implicit in what we do every day. Without it, there are significant roadblocks and things are less efficient, but only if you have clear communication amongst the whole team. Plans are more quickly seen through. Patient outcomes, I think, are better and it gives a feeling of the department working as a whole and not just individuals providing care.” Physician 5 - “I think the more communication errors I perceived are just the small ones that don’t affect the patient care directly but they affect relationships in the department. And again, it gets back to the trust and respect thing.”
5: Clinician gender and gender dyads influence communication dynamics, age and experience dyads did not.	Physician 2: “I think that males can be assertive without being perceived as bitchy. I think that female-to-female interaction between a … female team leader physician and … female nurse, I think that that dynamic actually can sometimes be more tense.” Nurse 3: “There’s so much more … casual sports conversations that go on between the male nurses and the male docs. And you see … so much more communication happen between them, even about … patients ... I’ve definitely had feelings of … like I can’t even talk to you right now, or I’m not part of this group right now”
6: Techniques for effective communication can be implemented.	Physician 3: “There are courses like TeamSTEPPS that can help with communication, I don’t know that they are specific to emergency medicine, though.”
