# Supplementary material for: Emergency Physician and Emergency Nurse Communication in the Emergency Department: A Mixed-methods Study
Source: West J Emerg Med. 2026 Jan 10;27(1):91–8. doi: 10.5811/westjem.48511 (PMC12815571; doi:10.5811/westjem.48511)
Supplement: Supplementary file 1 [file wjem-27-91-s001.pdf]

Appendix A-Literature Search

The survey was informed by topical literature review of existing healthcare communication literature within Ovid, Medline, CINAHL, PubMed using search terms Emergency Department, Emergency Medicine, emergency, nurse, physician, communication, nurse-physician relations, nurse-physician relations-evaluation, physician attitudes, multidisciplinary care teams, and interprofessional relations. Relevant articles were identified by study team members and reviewed for potentially relevant topic areas.

APPENDIX B

MD/RN Communication Survey

Q1. Are you a physician or nurse in an emergency department?
Yes
No

Q2. Do you work exclusively in pediatric emergency medicine?
Yes
No

Q3. How many years experience do you have working in emergency medicine (including training)?
Less than 2 years
2 or more years

Q4. Are you a physician or nurse?
Physician
Nurse

Q5. What is your sex?
Male
Female
Non-binary
Prefer not to answer
Prefer to self describe

Q6. Age
< 25 years old
25 - <35 years old
35 - <45 years old
45 - <55 years old

55+ years old

Q7. What is your race?
American Indian or Alaska Native
Asian
Black or African American
Native Hawaiian or Pacific Islander
White or Caucasion
Prefer not to identify

Q8. What is your ethnicity?
Hispanic or Latino
Non-Hispanic or Latino
Prefer not to identify

Q9. What emergency department do you work in for 50% or more of your clinical time?
xxx1
xxx2
xxx3
xxx4
Other (please specify)

Q10. How long have you been practicing emergency medicine (including training)?
More than 2 but less than 5 years
More than 5 but less than 10 years
More then 10 but less than 15 years
More than 15 but less then 20 years
More than 20 years

Q11. During an emergency department shift how often do you experience poor or ineffective physician-nurse communication?
Never
1-4 times per shift
5-10 times per shift
More than 10 times per shift

Q11. How often do you feel that poor or ineffective physician-nurse communication...

adversely affects patient care?
Never Sometimes Often Always

prevents the ED clinical team from functioning well?
Never Sometimes Often Always

affect the trust that you place in the individual nurse?
Never Sometimes Often Always

affect the trust that you place in most nurses?
Never Sometimes Often Always

Q19. How much are non-verbal behaviors a factor in effective team communication in the emergency department?
Never Sometimes Often Always

Q20. During an emergency department shift how often do you experience poor or ineffective physician-nurse communication?

Never

1-4 times per shift

5-10 times per shift

More than 10 times per shift

Q21. How often do you feel that poor or ineffective physician-nurse communication...
adversely affects patient care?
Never Sometimes Often Always

prevents the ED clinical team from functioning well?
Never Sometimes Often Always

affect the trust that you place in the individual physician/nurse?
Never Sometimes Often Always

affect the trust that you place in the most physicians/nurses?
Never Sometimes Often Always

Q22. How much are non-verbal behaviors a factor in effective team communication in the emergency department?
Never Sometimes Often Always

Q23. Can we contact you to participate in a focus group on nursing/physician communication in emergency medicine? We expect the focus group will last 1-2 hours, and you will get an Amazon Gift Card for participating.
Yes
No

Q24. Are you a nurse or a physician?
Nurse
Physician

Q25. What is the best email contact information for you?

Q26. Which times would work best for you to participate in a focus group?

Appendix C- Interview Guide

Interview Guide

For all of this discussion, when we say team, we are referring to the emergency physician and nursing team.

Goal: Establish importance of communication between nurses and physicians.

Say: Let's go around the room and each person say whether team communication is an important part of your job. In one or two sentences, please describe why or why not.

Goal: Identify needed and desired information exchange

Say: We are going to ask two questions about what you need and want to know from your colleague. Need is defined as information that you can't do your job without. Want is defined as information that you would like to know but could operate without.

In caring for a patient, what information do you feel you NEED to know from a physician/nurse to complete your job?

In caring for a patient, what information do you WANT to know from a physician/nurse to complete your job?

IF NEEDED: How do communication needs change depending on the situation?

Goal: Identify good communication techniques

Say: Think back to a situation in which communication between a physician and a nurse went well.

What aspects of communication worked well for patient care?

Why did that work well?

What aspects of communication worked well for the (team dynamic/physician-nurse) interactions?

Why did that work well?

Are there certain behaviors of a nurse/physician that seem to facilitate effective communication?

POSSIBLE FOLLOW UP IF TEAM DYNAMIC OR PATIENT CARE ARE NOT ADDRESSED:

Are there certain behaviors of a nurse/physician that seem to facilitate positive (team dynamic/patient care)?

Goal: Identify bad communication techniques

Say: Think back to a situation in which communication between a physician and a nurse went poorly.

What aspects of communication negatively affected patient care?

Why did that go poorly?

What aspects of communication negatively affected the (team dynamic/physician-nurse) interactions?

Why did that go poorly?

Are there certain behaviors of a nurse/physician that seem to facilitate poor communication?

POSSIBLE FOLLOW UP IF TEAM DYNAMIC OR PATIENT CARE ARE NOT ADDRESSED:

Are there certain behaviors of a nurse/physician that seem to facilitate negative (team dynamic/patient care)?

Goal: Assess how sex/age affect communication

How does the sex of the communicating parties affect communication?

How does age of the communicating parties affect communication?

Goal: Identify gaps in communication

Say: A communication gap is defined as information that would benefit patient care that is not delivered for one reason or another.

Are there communication gaps between doctors and nurses in the emergency department?

IF YES:

When do communication gaps occur?

Generally, what information is missed? **TRY TO KEEP THE RESPONSES BROAD**

Who commits communication gaps?

How could a system better support communication?

IF IT FOLLOWS:

What are the characteristics of the (clinicians/setting/patient) in which communication gaps occur?

Goal: Identify Behaviors that contribute to communication issues

Say: Non-verbal cues or behaviors are also an important part of communication.

What behaviors or non-verbal cues have you seen result in positive outcomes for patient care or team-dynamics?

What behaviors or non-verbal cues have you seen result in negative outcomes for patient care or team-dynamics?
